# Isolation, Identification, Sequence Analysis, and Pathogenicity of a CIAV Strain from *Aegypius monachus*

**DOI:** 10.1155/2023/6609077

**Published:** 2023-04-28

**Authors:** Mingrong Yin, Zhipeng Tang, Xiaoran Hu, Yuyan Li, Longzong Guo, Xiaolong Sun, Shuang Chang, Peng Zhao, Yixin Wang

**Affiliations:** ^1^College of Animal Science and Veterinary Medicine, Shandong Agricultural University, Tai'an, Shandong, China; ^2^Animal Disease Prevention and Control Center of Fushan District, Yantai, Shandong, China; ^3^Shandong Yisheng Livestock & Poultry Breeding Co., Ltd, Yantai, China

## Abstract

A cluster of vultures, artificially bred in a zoo in Shandong Province, China, displayed signs of emaciation and some even died, which raised the suspicion of an immunosuppressive pathogen. Upon conducting nucleic acid testing on the clinical samples, it was found that the CIAV was present in the tissue of dead vulture. In this study, samples of dead vulture tissue were used to isolate a chicken infectious anemia virus (CIAV) via the MDCC-MSB1 cell line, which was designated the SDTY2021-TJ strain. The full-length genomic sequence of SDTY2021-TJ was determined and analyzed in detail. The full genomic DNA of SDTY2021-TJ was found to be 2298 bp, with no omissions or additions in the coding region. The homology between the full-length genomic sequence of SDTY2021-TJ and the reference strain ranged from 95.5% (Del-Ros) to 98.7% (YN04). In comparison with the reference strain, the VP1 protein of SDTY2021-TJ contained a number of mutations. To assess the virulence of SDTY2021-TJ, one-day-old SPF chickens were inoculated with both high and low doses of the pathogen. The results demonstrated that SDTY2021-TJ had a considerable pathogenicity to SPF chickens, as the high-dose group caused a 50% mortality rate, and even the low-dose group caused a 30% mortality rate. The chickens infected with the disease exhibited paleness in the cockscomb and stunted growth, as well as a compromised response to NDV and AIV-H9 inactivated vaccine. Furthermore, histological observation revealed an atrophy and degeneration of the thymus. To the best of our knowledge, this is the first instance of CIAV being isolated from artificially bred *Aegypius monachus*, implying that wild birds may be involved in the transmission and spread of CIAV.

## 1. Introduction

Chicken infectious anemia (CIA) is a disease caused by the chicken infectious anemia virus (CIAV) and is identified by anemia and the shrinking of the thymus [1, 2]. CIAV is mainly found to infect chicks of a low age, especially the newly born chicks. Given the fact that the clinical manifestations of CIAV are usually uncommon, breeders of feeders often overlook the occurrence of CIAV. Chickens infected with CIAV are subject to immunosuppression, making them more prone to other poultry pathogens, including viruses, bacteria, and parasites [2]. Infection with CIAV can heighten the virulence of attenuated vaccines or diminish the efficacy of some vaccines, such as Marek's disease virus (MDV) vaccine, thus resulting in the ineffectiveness of the vaccination [3, 4]. Since its first isolation in Japan, CIAV has been widely prevalent worldwide [5–11]. CIAV is able to spread in a vertical or horizontal manner, remaining resilient in chicken flocks and posing a challenge to be eliminated, causing major financial losses to the global poultry breeding industry [12].

CIAV, belonging to the *Circoviridae*, the genus *Gyrovirus*, family *Anelloviridae*, is a small DNA virus with an icosahedral capsid about 20 nm diameter in size [13]. The CIAV genome, with a length of either 2298 or 2319 nucleotides, is composed of three overlapping open reading frames, which are translated into three major viral proteins: VP1 (51.6 kDa), VP2 (24 kDa), and VP3 (13.6 kDa). VP1, the capsid protein, is a vital structural protein of the virus; VP2, a nonstructural protein, is essential for the virus assembly process acting as a scaffold [14]; VP3, also known as apoptin, is a viral nonstructural protein which has an effect on pathogenesis, inducing apoptosis in infected cells and some tumor cells [15, 16]. Analysis of the noncoding region reveals a signal region for polycondensation and a regulatory region for virus replication [17]. Sequence alignment results demonstrate minimal gene sequence variations between different CIAV strains [18].

CIAV can be spread from one chicken to another through direct contact, or through indirect contact with materials that have been contaminated, such as feed and water. It is widely accepted that chickens are the primary carriers of CIAV, although other avian species may also contribute to its transmission. In 1998, research revealed that CIAV was not only capable of infecting chickens but also quail, pigeon, and crow [19]. An investigation of 1199 wild bird samples from Northeast China revealed a positive rate of 5.34% for CIAV, suggesting that wild birds may be implicated in the transmission of CIAV. More importantly, it is possible that cats and dogs may be a vector for the transmission of CIAV, as genomic fragments of the virus were detected in the feces of some mammals [20].

Our laboratory received two specimens from a zoo located in Shandong Province, China in 2021, which were of diseased vultures artificially bred there. These vultures were given food in a relatively enclosed environment. The ill vultures displayed signs of depression and died at irregular intervals, and it was suspected that they had been infected by an immunosuppressive pathogen. By utilizing PCR testing, the veterinarian at the zoo had already eliminated the probability of influenza virus, Newcastle disease virus, and other typical pathogenic infections. This study initially identified immunosuppressive pathogens, including avian leukosis virus (ALV), reticula endotheliosis virus (REV), MDV, and CIAV. PCR detection identified the presence of CIAV, which was then isolated by inoculating the MDCC-MSB1 cell line. Subsequently, the genome of this vulture-originated CIAV strain was fully determined and its virulence to SPF chickens was evaluated.

## 2. Materials and Methods

### 2.1. Samples and Cells

Specimens of the liver and spleen were taken from a dead vulture from a zoo in Shandong Province, China. MDCC-MSB1 cells were purchased from ATCC and stored in our laboratory.

### 2.2. PCR Detection of Clinical Samples

DNA from vulture clinical samples was extracted using the TIANamp Genomic DNA Kit (Tiangen Biotechnology Co., Ltd., China) in accordance with the manufacturer's instructions. The PCR technique was employed to detect ALV, REV, MDV, and CIAV; the sequence of primers is provided in Table 1.

### 2.3. Virus Isolation

Based on the results of PCR detection, the CIAV was isolated through the utilization of the MDCC-MSB1 cell line according to the references in Reference [21]. Liver and spleen tissues were homogenized with PBS buffer, subjected to freezing and thawing cycles thrice, and centrifuged for 2 minutes at 12000 rpm. A 0.22 *μ*M filter was used to sterilize the sample suspensions, which were then introduced into MDCC-MSB1 cells for 1 hour. Following this, the medium was switched and the cells were cultured for 5–7 days. The infected cells were harvested and freeze-thawed three times, and the supernatant was used to inoculate fresh MDCC-MSB1 cells. Following three generations of continuous passaging, supernatant was collected when the cells exhibited clear signs of pathology and stored at −80°C. To verify the isolation of CIAV, PCR amplification and indirect immunofluorescence assay (IFA) were employed, and the resulting strain was designated SDTY2021-TJ.

### 2.4. IFA Assay

MDCC-MSB1 cells were subjected to infection with SDTY2021-TJ viral stock. After two days, the cells were washed twice with PBS and subsequently fixed with 4% paraformaldehyde for 10 minutes. The CIAV VP1 monoclonal antibody was initially incubated at 37°C for 45 minutes and then followed by the addition of FITC-labeled goat antimouse fluorescent antibody, which was also incubated at 37°C for 45 minutes. Finally, the cells were stained with DAPI for 5 minutes and then viewed using the confocal microscope.

### 2.5. CIAV Genomic DNA Sequencing

Following the manufacturer's instructions, the DNA of SDTY2021-TJ viral stock was extracted using the TIANamp Genomic DNA Kit (Tiangen Biotechnology Co., Ltd., China) and PCR was then used to amplify the full-length of the genomic sequence. Primers were designed according to the Cux-1 sequence (accession no. M55918), and the sequence is presented in Table 1. PCR was performed in a 25 *μ*l reaction volume containing 0.5 *μ*l forward primers and downstream primers (10 mM), 1 *μ*l DNA, 2.5 *μ*l buffer, 2 *μ*l dNTPs (2.5 mM), 0.5 *μ*l high fidelity DNA polymerase (TaKaRa Bio, Inc., Dalian, China), and 18 *μ*l deionized water. The PCR reaction conditions were as following: 98°C predenaturation for 1 min, followed by 98°C denaturation for 10 s, 56°C annealing for 1 min, and 72°C extension for 30 s; the extension was carried out for another 10 minutes before terminating the reaction at 4°C. The PCR products were purified and then sequenced, and the full-length of CIAV genomic sequence was assembled using the SeqMan program from the DNASTAR software package (Madison, WI, USA).

### 2.6. Sequence Alignment and Phylogenetic Analysis

The genomic sequences of SDYT2021-TJ and reference CIAV strains downloaded from GenBank were analyzed using DNASTAR software. The phylogenetic analysis was achieved through a bootstrap analysis of 1000 replicates with the help of neighbor-joining method and the MEGA 7.0 software program. The reference CIAV strains downloaded from GenBank were listed in Table 2.

### 2.7. Animal Experiment Design

To assess the pathogenicity of SDYT2021-TJ, sixty-one-day-old leghorn specific pathogen-free (SPF) chickens (SAIS Poultry Co., Jinan, China) were randomly allocated into three groups, each with twenty chickens. On the first day, the low-dose group was injected with 300 EID_50_ SDTY2021-TJ intramuscularly, the high-dose group was inoculated with 1000 EID_50_ SDTY2021-TJ intramuscularly, and the control group was administered PBS of equal volume. The animal experiment ran for a period of 35 days. During this period, chicken flocks were observed daily, body weights were measured weekly, and the mortality rate was determined at the end of the experiment. At 14 days of age, three chickens from each group were sacrificed and their thymuses were collected to prepare paraffin sections for histopathological examination.

To investigate the effect of SDTY2021-TJ infection on red blood cells, anticoagulants were collected at 1 w, 2 w, and 3 w, and the PE-6800vet automatic blood cell analyzer (IDEXX, USA) was employed to determine the number of red blood cells and hematocrit (Hct). In order to evaluate the immunosuppressive effects of SDTY2021-TJ on response to NDV and AIV-H9 vaccination, chickens belonging to all groups were administered subcutaneous injections of inactivated NDV (Qilu Animal Health Co., Jinan, China) and AIV-H9 (Qilu Animal Health Co., Jinan, China) at 1-day-old. The hemagglutination inhibition (HI) assay was employed to measure NDV and H9-AIV titers in serums collected at 1, 2, and 3 weeks [22].

### 2.8. Data Statistical Analysis

All the results are presented as the Means ± SEMs. All statistical analyses were performed using SPSS statistical software package for Windows, version 17.0 (SPSS Inc., Chicago, USA). The data were analyzed by one-way ANOVA followed by Duncan's multiple comparison to determine the differences of body weight and antibody response to NDV and AIV-H9 between different groups.

### 2.9. Ethics Statements

The animal experiments were approved by Shandong Agricultural University Animal Care and Use Committee. The license number was SDAU-2022-004. Care and maintenance of all chickens were in accordance with the guidelines of the Committee on the Ethics of Animal of Shandong Agricultural University and the biosecurity guidelines.

## 3. Results

### 3.1. Isolation and Identification of CIAV

**Figure 1:**
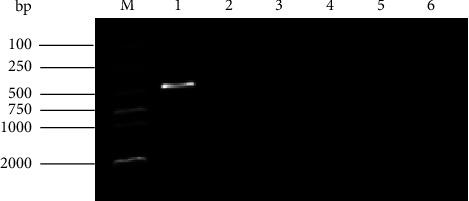
PCR detection of immunosuppressive viruses in clinical samples M: 2,000 Marker; 1: CIAV; 2: ALV-A; 3: ALV-B; 4: ALV-J; 5: REV: 6: MDV.

**Figure 2:**
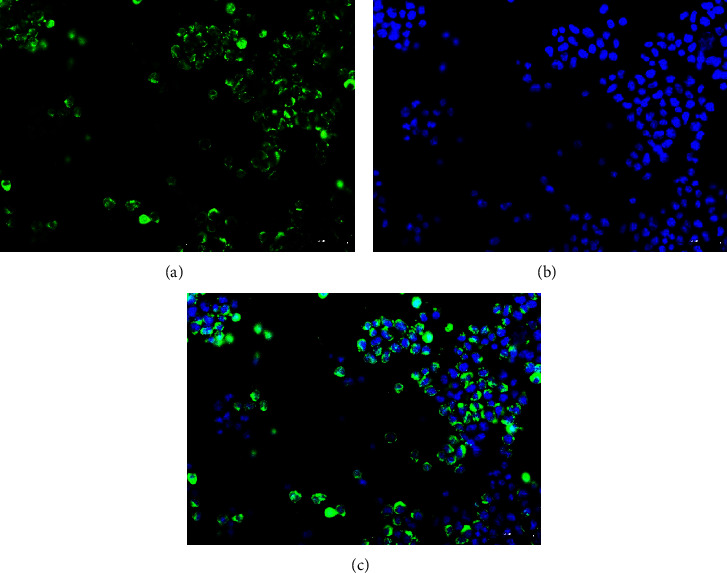
IFA detection of MDCC-MSB1 cells infected with SDTY2021-TJ (a) detect with VP1 monoclonal antibody; (b) DAPI staining; (c) merge of (a) and (b).

To ascertain whether vultures were infected by immunosuppressive virus, we conducted a PCR test on the dead vulture samples. The PCR analysis indicated CIAV DNA was present, whereas no ALV, REV, or MDV DNA was detected. By inoculating MDCC-MSB1 cells with sample suspensions for three generations, the PCR detection on the cellular supernatant demonstrated a successful isolation of a CIAV strain, which was designated as SDTY2021-TJ (Figure 1). To further validate the isolation of SDTY2021-TJ, IFA was conducted using CIAV VP1 monoclonal antibody. The results indicated that the cytoplasm of infected MDCC-MSB1 cells displayed a green positive fluorescence staining, whereas there was no fluorescence signal observed in uninfected cells (Figure 2).

### 3.2. Characteristics of Full-Length Sequence of SDTY2021-TJ

**Figure 3:**
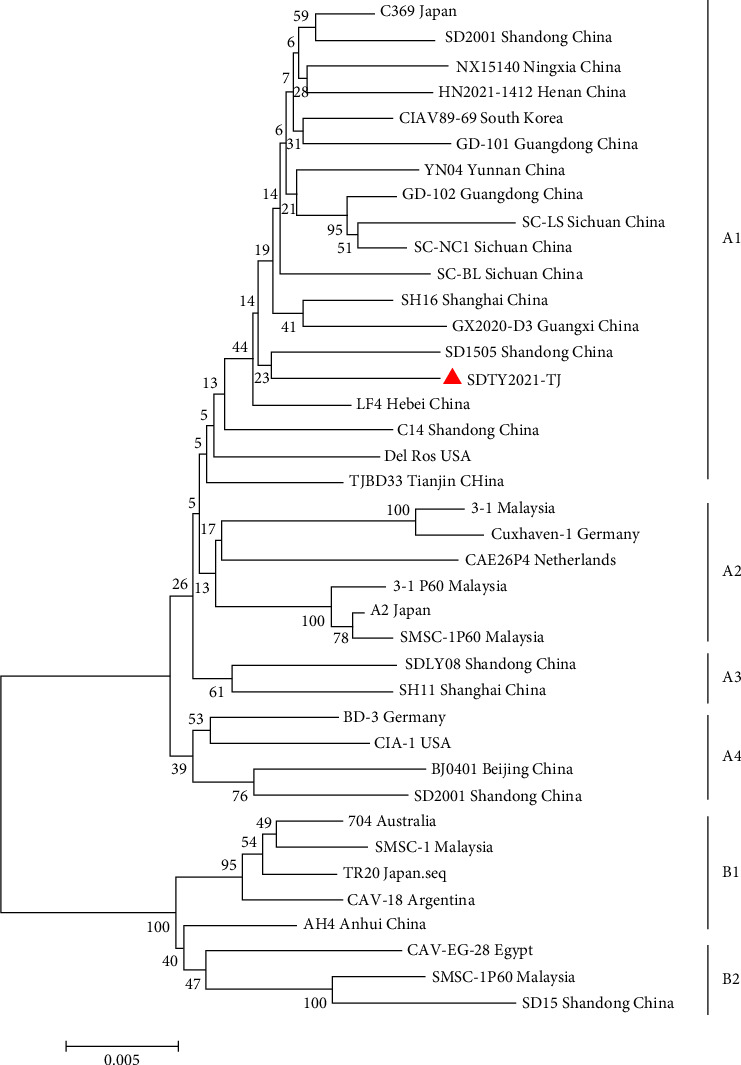
Phylogenetic tree of full-length genome sequences of SDTY2021-TJ and reference CAV strains. The phylogenetic analysis was carried out by neighbor-joining method by a bootstrap analysis of 1000 replicates using MEGA 7.0 software program. SDTY2021-TJ was labeled with red triangle.

Through PCR amplification, the full-length sequence of SDTY2021-TJ was obtained. SDTY2021-TJ was identified to have a genome length of 2298 bp, which contained three ORFs encoding VP1, VP2, and VP3, without any insertions or deletions in its coding region. Its genome sequence was subsequently uploaded to the GenBank database. The full-length sequence of SDTY2021-TJ was compared to the reference strain, showing homology levels between 95.5% (Del-Ros) and 98.7% (YN04). Results from the genetic evolution of the complete genome of the CIAV virus show that it can be divided into two distinct groups, A and B. Group A contains four genotypes, while Group B holds one [18]. SDTY2021-TJ belongs to the first genotype of Group A (Figure 3). To determine if the genome of SDTY2021-TJ had undergone recombination, several methods in the software suite of Recombination Detection Program 4 (RDP v.4.97) were used to identify the hypothetical recombination events [23]. However, we did not find any possible reorganization events in SDTY2021-TJ.

### 3.3. Molecular Characterization of SDTY2021-TJ VP1 Sequences

**Figure 4:**
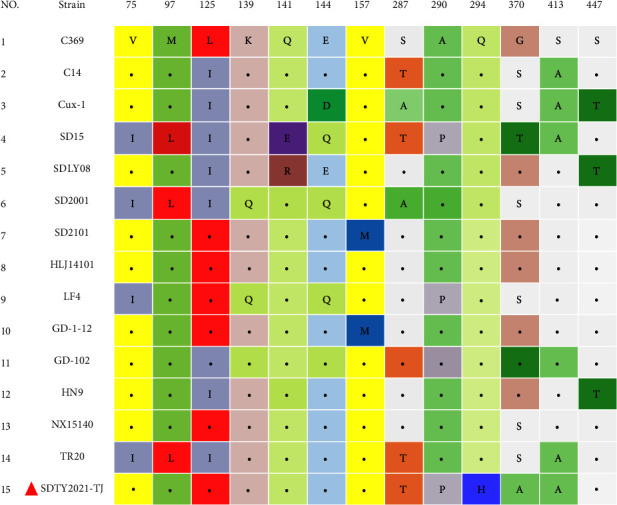
Amino acid substitutions observed in SDTY2021-TJ isolated in this study.

The nucleotide sequence of SDTY2021-TJ and the reference strain VP1 exhibited homology levels ranging from 95.3% to 98.7%. The amino acid sequence of the isolate VP1 had the lowest homology with CAV-EG-28 (95.3%) and the highest homology with YN04 (98.7%). A comparison of the amino acid sequence of the isolate VP1 to that of the reference strain VP1 revealed mutations at positions 75, 97, 125, 139, 141, 144, 157, 251, 287, 290, 294, 370, 413, and 447. Specifically, these mutations included V157M, S287T, A290P, Q294H, and G370A (Figure 4).

### 3.4. Pathogenicity Investigation of SDTY2021-TJ

#### 3.4.1. Effects of SDTY2021-TJ on Mortality and Body Weight of SPF Chickens

**Figure 5:**
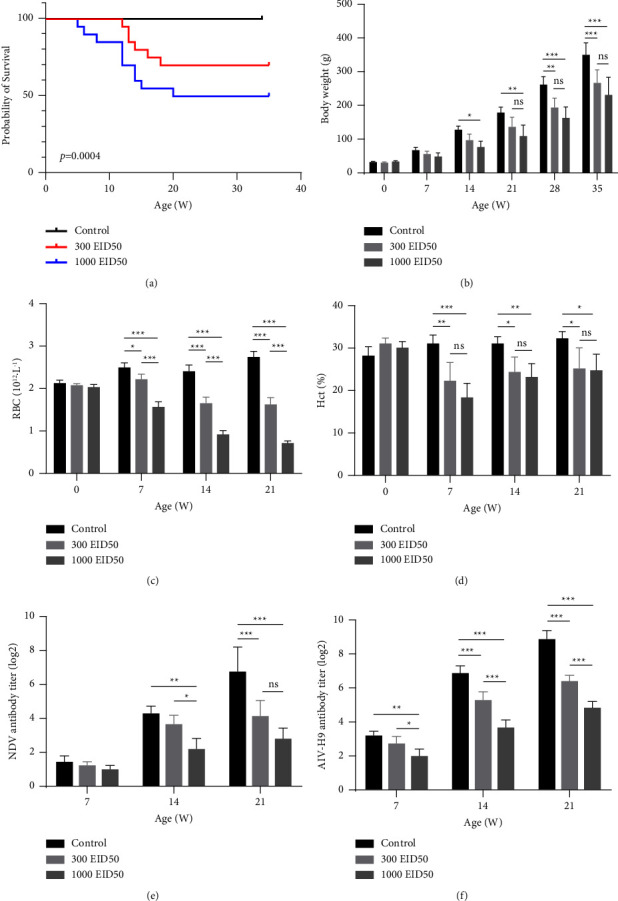
Pathogenicity analysis of SDTY2021-TJ on SPF chickens: (a) survival curves of chickens in different groups; (b) body weight gain of chickens in different groups; (c) red blood cell numbers of chickens in different groups; (d) Hct of chickens in different groups; (e) antibody titers of chickens in different groups after immunization with NDV inactivated vaccine; (f) antibody titers of chickens in different groups after immunization with AIV-H9 inactivated vaccine. Differences were considered significant difference when *P* ≤ 0.05 (^*∗*^), highly significant when *P* ≤ 0.01 (^*∗∗*^) and extremely significant *P* ≤ 0.001 (^*∗∗∗*^). The error bars represent the SEM. ns, no significant difference (*P* > 0.05).

The chickens in the low-dose group began to perish on the 12th day, with six of them (6/20) succumbing in succession, the peak of mortality occurring between the ages of thirteen and fifteen days. The chickens in high-dose group began to perish on the 5th day, with ten of them (10/20) succumbing in succession, the peak of mortality occurring between the ages of twelve and fourteen days (Figure 5(a)). The infected chickens exhibited a depressed state, were smaller and of varied sizes, had drab and dull feathers, and pallor on their crowns and feet. The body weight of chickens in the low-dose group was significantly reduced in comparison to the control group at 28 and 35 days, while the body weight of chickens in the high-dose group was significantly lower at 14 days of age (Figure 5(b)), and this disparity continued to grow with age, demonstrating that SDTY2021-TJ had a considerable impact on their growth performance. The body weight of the high-dose group was not significantly different from that of the low-dose group.

#### 3.4.2. Effect of SDTY2021-TJ on Erythrocytes of SPF Chickens

To evaluate the effects of SDTY2021-TJ on chickens infected with different doses, we examined the number of erythrocytes and Hct as CIAV is known to cause anemia in chickens (Figures 5(c) and 5(d)). The results indicated that the erythrocyte count and Hct of the infected chickens at 7, 14, and 21 days of age were significantly lower than those of the control group, and the number of erythrocytes in the high-dose group was significantly less than that of the low-dose group, but there was no significant statistical difference between the two doses in terms of Hct.

#### 3.4.3. Effect of SDTY2021-TJ on the Response of Vaccination in SPF Chicken

To evaluate the immunosuppressive effect of SDTY2021-TJ, 1-day-old chickens were immunized using NDV and AIV-H9 vaccines, and the antibody levels of NDV and AIV-H9 were monitored on days 7, 14, and 21. It was observed that the NDV antibody titer of chickens in the high-dose group was significantly reduced compared to the control group on the 14th day, and the NDV antibody titer of chickens in the low-dose group was lower than the control group on the 21st day (Figure 5(e)). The chickens in the high-dose group had significantly lower H9 antibody titers on the 7th day postimmunization compared to the control group, and the inhibition of CIAV infection was more pronounced for H9 antibody than for NDV antibody (Figure 5(f)).

#### 3.4.4. Effect of SDTY2021-TJ on Thymus Structure of SPF Chicken

**Figure 6:**
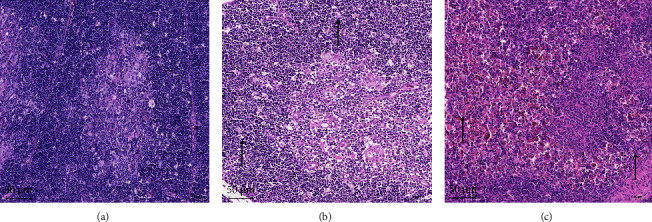
Section observation of chicken thymus in different groups after CIAV challenge: (a) chicken thymus of control group (100×); (b) chicken thymus of low-dose group (100×); (c) chicken thymus of high-dose group (100×).

The examination revealed that the thymus, bursa of Fabricius and spleen had undergone atrophy, and the bone marrow was a pale yellow hue. Subsequently, the chicken thymus in each group was collected and prepared for HE section, which was then observed under a microscope. The results demonstrated that the thymus of chickens in the control group had a normal structure, with a distinct boundary between the cortex and medulla and the cells closely arranged (Figure 6(a)). In the low-dose group, the thymus tissue structure was slightly abnormal, with a dark cortex and light medulla visible in the field of vision (Figure 6(b)). In the high-dose group, the thymus structure showed moderate abnormality, with the dark cortex in the peripheral and peripheral visual field largely atrophied, and a decreased number of small lymphocytes in the cortex (Figure 6(c)).

## 4. Discussion

CIA is an important infectious disease of poultry, which seriously affects poultry breeding around the world [24]. A major consequence of CIA on chickens is the weakening of their immune system, thus resulting in greater vulnerability to other viruses and bacteria, thus making the symptoms more intricate. The pathogen of CIA, CIAV was first identified in a contaminated chicken Marek's disease vaccine and then spread worldwide [11]. Chickens of all ages and breeds are vulnerable to CIAV; especially chickens of 1-day-old are the most vulnerable. This virus has been identified in poultry all over the world, causing severe damage to the poultry industry [2].

Previous epidemiological investigations largely focused on poultry, with limited data available on wild bird infection. Researchers have determined that CIAV can infect quail, pigeon, crow, as well as certain wild birds, signifying that these species may be involved in the transmission of the disease [19]. The expansive living environment of wild birds, coupled with their yearly migratory behavior, could potentially facilitate the rapid and widespread spread of the virus creating a more challenging situation for prevention and control efforts.

In 2022, our laboratory received two samples of vultures that had been artificially fed in a zoo, displaying symptoms of emaciation, depression, and sporadic death. PCR analysis of the samples confirmed the presence of CIAV, while no other viruses such as ALV, REV, and MDV were detected. To eliminate the possibility that vultures may be affected by any other unknown pathogens, we performed high-throughput sequencing of RNA from the samples and the results verified that only CIAV was present in clinical samples (data not shown). Subsequently, the CIAV, designated SDYT2021-TJ, was successfully isolated through inoculation of sample suspensions into MDCC-MSB1 cells. This was the first discovery that artificially bred vultures could infect with CIAV, which is in line with the findings of previous studies that wild birds can spread the virus.

To investigate the sequence features of SDYT2021-TJ, we analyzed its genome sequence in comparison to other reference strains. The gene sequence analysis revealed that the full-length genome of SDTY2021-TJ was highly homologous to that of chicken-origin CIAV, with the nucleotide homology ranging from 95.9% (SD15) to 99.1% (JL14023). The zoo's keeper stated that the vultures that were artificially fed had not been in contact with any outside birds, but were given chicken meat products. Consequently, it is possible that the vultures infected with CIAV were exposed to chicken meat products that were infected or contaminated with chicken-origin CIAV. We conducted a further investigation into the features of the virus VP1 protein. Previous investigations have revealed that amino acid positions in the CIAV VP1 protein, including 75, 89, 125, 139, 141, 144, and 394, are related to virus replication and pathogenicity [25, 26]. It has been established that the substitution of 139 and 144 amino acids will have an effect on the replication and virulence of the virus, which is also linked to the rate of its spread. The genome of SDTY2021-TJ was found to have T89, Q141, K139, E144, and Q394, suggesting that it could replicate quickly *in vitro* and had a high pathogenicity to chickens. Furthermore, mutations in the VP1 amino acid of SDTY2021-TJ were observed, yet the biological implications of these mutations still require further investigation through reverse genetics assay.

CIAV, being an immunosuppressive virus, can lead to growth retardation, heightened mortality, and an increase in the occurrence of secondary and multiple infections in chickens [27, 28]. To further assess the pathogenicity of this strain to chickens, an animal experiment was conducted in this research. We observed that SDTY2021-TJ when administered to 1-day-old SPF chickens in both low and high doses caused morbidity and mortality; the high-dose resulted in 50% mortality, while the low-dose caused 30% mortality. The chicken which was ill displayed a faint crest, an inhibited increase in weight, and a drastic decrease in the number of red blood cells. The autopsy revealed that the thymus of the ailing chicken was atrophic and deteriorated, and the HE section showed that the thymocytes had experienced obvious apoptosis. Results have demonstrated a significant decrease in the immune response of chickens infected with NDV and AIV-H9 inactivated vaccines. The data revealed that SDTY2021-TJ had a significant pathogenicity. As the age of the infected chickens increased, the mortality rate decreased and the weight of the chickens eventually recovered, which is in accordance with existing literature that suggests that CIAV is highly pathogenic to chicks, yet the chickens' resistance to CIAV increases over time. Regrettably, the study was unable to determine the pathogenicity of CIAV to vultures as a result of vultures being protected animals in China.

In summary, the first-ever CIAV strain was isolated from vultures in the present investigation and its full-length genome, along with its pathogenicity, was characterized and analyzed. This research determined that vultures can be carriers of chicken CIAV and that contaminated food may be the means of transmission. It is essential to take into account the role of wild birds in the prevention and control of this virus.

## Figures and Tables

**Table 1:** Sequences of primers used in this study.

No.	Primers	The sequences of the primers (5′ ⟶ 3′)	Sizes (bp)
1	CIAV-F	AGAAAGTCAAGATGGACGAATC	440
	CIAV-R	ATCTTCTTGGAGAGCGTTCAT	

2	ALVA-F	CGGAGAAGACACCCTTGCT	715
	ALVA-R	GCATTGCCACAGCGGTACTG	

3	ALVB-F	CGGAGAAGACACCCTTGCT	515
	ALVB-R	GTAGACACCAGCCGGACTATC	

4	ALVJ-F	CGGAGAAGACACCCTTGCT	422
	ALVJ-R	CGAACCAAAGGTAACACACG	

5	REV-F	CATACTGGAGCCAATGGTT	320
	REV-R	AATGTTGTAGCGAAGTACT	

6	MDV-F	GCCTTTTATACACAAGAGCCGAG	560
	MDV-R	TTTATCGCGGTTGTGGGTCATG	

7	CIAV-com-F1	GCATTCCGAGTGGTTACTATTCC	842
	CIAV-com-R1	CGTCTTGCCATCTTACAGTCTTAT	

8	CIAV-com-F2	CGAGTACAGGGTAAGCGAGCTAAA	990
	CIAV-com-R2	TGCTATTCATGCAGCGGACTT	

9	CIAV-com-F3	ACGAGCAACAGTACCCTGCTAT	802
	CIAV-com-R3	CTGTACATGCTCCACTCGTT	

*Note*. Primers 1–6 were used to detect viral DNA in clinical samples; primers 7–9 were used to amplify the full-length of CIAV genome.

**Table 2:** Detailed information of CIAV reference strains used in this study.

Strains	Time	Country	Accession no.
CIA-1	1999	USA	L14767
Del-Ros	2000	USA	AF313470
SMSC-1	2003	Malaysia	AF285882
SMSC-1P60	2003	Malaysia	AF390102
Cux-1	2008	Netherlands	M55918
CAE26P4	2007	Netherlands	D10068
Cuxhaven 1	1992	Germany	M81223
BD-3	2004	Germany	AF395114
TR20	1999	Japan	AB027470
A2	2000	Japan	AB031296
C369	2001	Japan	AB046590
CAV-EG-28	2018	Egypt	MH001570
CAV-18	2014	Argentina	KJ872514
CIAV89-69	2013	South Korea	JF507715
BJ0401	2004	Beijing, China	DQ124934
TJBD33	2005	Tianjin, China	AY843527
LF4	2005	Tianjin, China	AY839944
AH4	2005	Anhui, China	DQ124936
SH11	2005	Shanghai, China	DQ141670
SH16	2005	Shanghai, China	DQ141671
GD-101	2013	Guangdong, China	KU050680
GD-102	2013	Guangdong, China	KU050677
SC-LS	2014	Sichuan, China	KM496304
SC-BL	2014	Sichuan, China	KM496300
SC-NC1	2014	Sichuan, China	KM496308
C14	2004	Shandong, China	EF176599
SDLY08	2008	Shandong, China	FJ172347
SD15	2015	Shandong, China	KX811526
SD1505	2015	Shandong, China	KU645523
SD2001	2020	Shandong, China	OL448832
SD2101	2021	Shandong, China	OL448842
YN04	2020	Yunan, China	MZ540762
GX2020-D3	2020	Guangxi, China	MW579761
HN2021-1412	2021	Henan, China	MZ369153

## Data Availability

The data used to support the findings of this study are available from the corresponding author upon request.
